# Calcium adsorption and displacement: characterization of lipid monolayers and their interaction with membrane-active peptides/proteins

**DOI:** 10.1186/1471-2091-7-15

**Published:** 2006-05-03

**Authors:** Sven O Hagge, Malte U Hammer, Andre Wiese, Ulrich Seydel, Thomas Gutsmann

**Affiliations:** 1 Research Center Borstel, Leibniz-Center for Medicine and Biosciences, Department of Immunochemistry and Biochemical Microbiology, Division of Biophysics, Parkallee1-40, D-23845 Borstel, Germany

## Abstract

**Background:**

The first target of antimicrobial peptides (AMPs) is the bacterial membrane. In the case of Gram-negative bacteria this is the outer membrane (OM), the lipid composition of which is extremely asymmetric: Whereas the inner leaflet is composed of a phospholipid mixture, the outer leaflet is made up solely from lipopolysaccharides (LPSs). LPS, therefore, represents the first target of AMPs. The binding and intercalation of polycationic AMPs is driven by the number and position of negatively charged groups of the LPS. Also, proteins other than cationic AMPs can interact with LPS, e.g. leading eventually to a neutralization of the endotoxic effects of LPS. We compared different biophysical techniques to gain insight into the properties of the electrical surface potentials of lipid monolayers and aggregates composed of LPSs and various phospholipids and their interaction with peptides and proteins.

**Results:**

The net negative charge calculated from the chemical structure of the phospholipid and LPS molecules is linearly correlated with the adsorption of calcium to two-dimensional lipid monolayers composed of the respective lipids. However, the ζ-potentials determined by the electrophoretic mobility of LPS aggregates can only be interpreted by assuming a dependence of the plane of shear on the number of saccharides and charged groups. Various peptides and proteins were able to displace calcium adsorbed to monolayers.

**Conclusion:**

To characterize the electrical properties of negatively charged phospholipids and LPSs and their electrostatic interaction with various polycationic peptides/proteins, the adsorption of calcium to and displacement from lipid monolayers is a suitable parameter. Using the calcium displacement method, the binding of peptides to monolayers can be determined even if they do not intercalate. The interpretation of ζ-potential data is difficulty for LPS aggregates, because of the complex three-dimensional structure of the LPS molecules. However, the influence of peptides/proteins on the ζ-potential can be used to characterize the underlying interaction mechanisms.

## Background

Cell membranes composed of lipids and proteins are an essential part of all living organisms. The interaction between peptides or proteins and lipid membranes is driven by entropic effects and in many cases also by electrostatic forces. The architecture of membranes differs for various cell types and with that also the specificity of the interaction between lipids and peptides/proteins.

The cytoplasmic membrane of mammalian cells has an asymmetric lipid distribution [1], in particular phosphatidylserine (PS) is almost exclusively located in the inner leaflet of the membrane, and in the early steps of apoptotic cell death it is translocated into the outer leaflet [2]. It has been shown that an increased amount of PS in the outer leaflet, e.g. in cancer cells, can lead to increased binding of the polycationic NK-lysine derived peptide NK-2 and a preferential killing of these cells [3].

The important influence of electrostatic interactions is even more pronounced for the interaction between bacteria and antimicrobial peptides (AMPs) of the innate host defense system. Membranes of Gram-positive as well as of Gram-negative bacteria express a high amount of negatively charged lipids on the outer leaflet of the membrane which is in direct contact with the extracellular environment.

**Figure 1:**
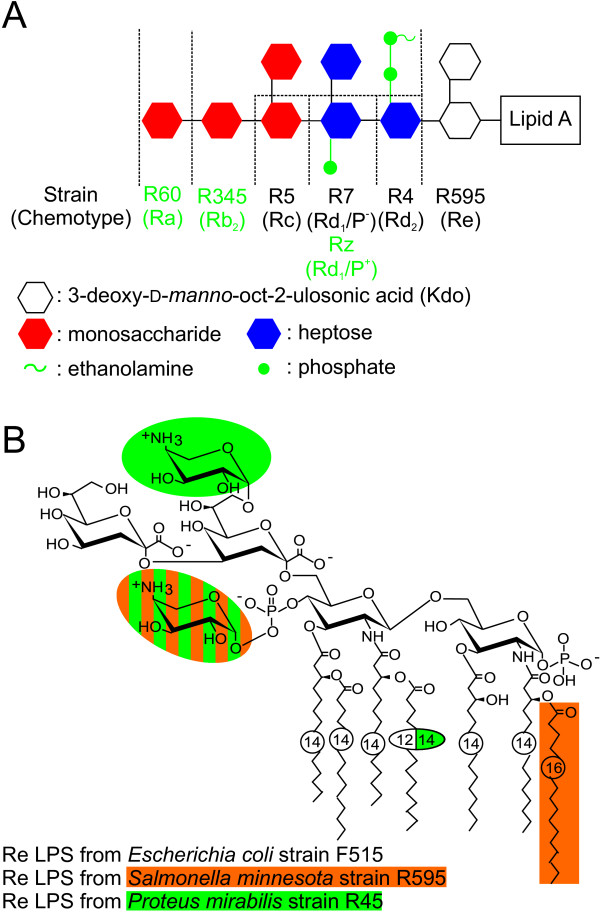
**Chemical structures of the LPSs used**. (A) Schematic chemical structure of the core region of LPSs from different rough mutant strains of *Salmonella minnesota *and its chemotypes (Ra to Re). The phosphate residue at the second heptose and the 2-aminoethyl diphosphate residue at the first heptose are only present in the mutants Rz, R345 and R60. (B) Chemical structure of Re LPSs from various Gram-negative species, i.e., *Escherichia coli *strain F515, *Salmonella minnesota *strain R595, and *Proteus mirabilis *strain R45.

Gram-positive bacteria have a cytoplasmic membrane containing negatively charged diphosphatidylglycerol (DPG, cardiolipin) [4] and lipoteichoic acid (LTA) [5] on the outer leaflet. The cell envelope of Gram-negative bacteria is composed of two membranes, the inner and the outer membrane. The inner leaflet of the outer membrane is composed of phospholipids and the outer leaflet of glycolipids, in most cases lipopolysaccharides (LPSs, endotoxin) [6]. LPS is negatively charged due to phosphate groups linked to the di-glucosamin sugar backbone and to the 3-deoxy-alpha-D-manno-oct-2-ulosonic acids (Kdo's) and further negatively charged residues (Fig. 1). This highly negatively charged surface is the first target for a number of polycationic AMPs, e.g. defensins [7], cathelicidins [8], bactericidal/permeability increasing protein (BPI) [9], and magainins [10]. Many AMPs and other polycationic peptides have a dual function, they are antimicrobial and they can reduce or even inhibit the LPS-induced activation of human mononuclear cells. A reduction of the LPS-induced cytokine release has been shown for the endotoxin-neutralizing protein (ENP) [11], polymyxin B [12], lysozyme [13], peptides based on Limulus anti-lipopolysacchride factor [14], and a number of further peptides and proteins. Moreover, the charges of LPS are important for the interaction between LPS and proteins which are involved in the LPS-induced transmembrane signal transduction, e.g. the LPS-binding protein [15], leading to the release of cytokines (for review see [16]).

To investigate the interactions between negatively charged lipids and polycationic peptides/proteins on a molecular level, the knowledge of the number of charges per lipid molecule or the surface charge density of the lipid aggregates is of importance. A number of techniques to determine these parameters is available. Some of the methods can be applied for whole cells or bacteria (for review see [17]), for lipid aggregates, for planar lipid bilayers, or for lipid monolayers.

Four different methods to determine values which depend on the number of charges per molecule and the size of the molecules are compared in this paper for phospholipids and, in particular, for LPS:

(i) Calculation of the net charge per molecule which is based on the chemical structure as determined in mass spectrometric experiments.

(ii) Determination of innermembrane potentials of monolayers [18,19] or planar lipid bilayers [20,21].

(iii) Determination of the electrophoretic mobility of cells and lipid aggregates. From the electrophoretic mobility, the ζ-potential can be calculated which is correlated with the surface charge density [17].

(iv) Utilization of marker molecules, which bind to specific lipids, e.g. annexin V as a marker for PS in apoptotic cells, or to charged residues, e.g. calcium doped with small amounts of the β-active isotope ^45^Ca^2+ ^as a marker for negatively charged lipids.

**Figure 2:**
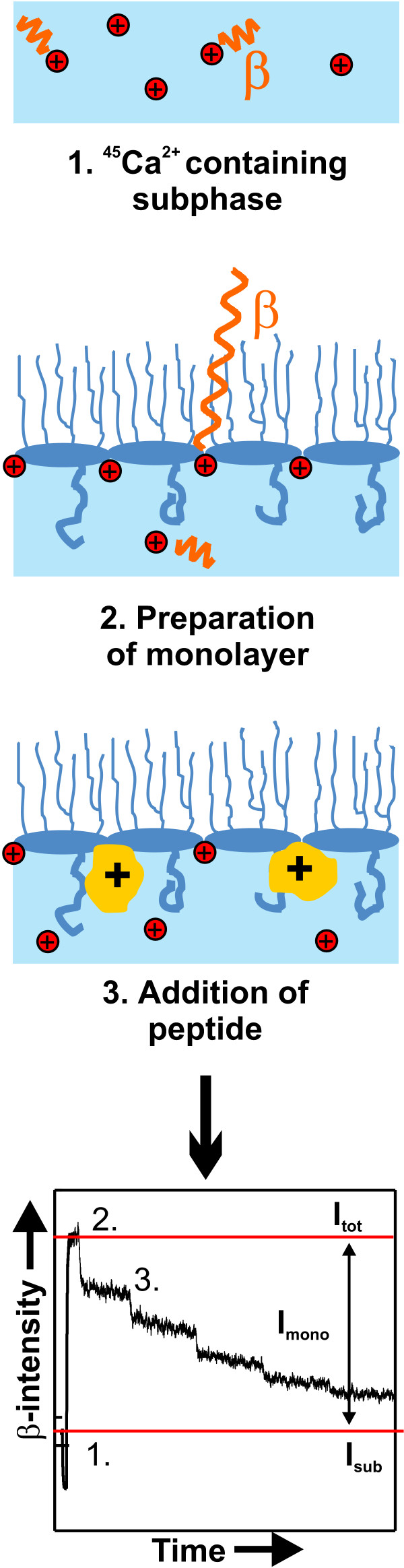
**The calcium displacement technique**. The β-radiation emitted from ^45^Ca^2+ ^ions in an aqueous subphase is shielded by the aqueous layer above the ions, yielding the β-intensity *I*_*sub*_. (2.) A monolayer prepared on the subphase leads to the adsorption of calcium and in particular of ^45^Ca^2+ ^ions to the surface, and subsequently a higher β-radiation *I*_*tot *_can be detected. The β-intensity resulting from the monolayer can be calculated as *I*_*mono *_= *I*_*tot *_- *I*_*sub*_. (3.) Substances having a higher affinity to the lipid monolayer as compared to Ca^2+ ^lead to the displacement of the Ca^2+ ^ions from the surface and a reduction of the β-intensity *I*_*tot*_. The titration of peptides/proteins into the buffer can lead to a time trace of the β-intensity as depicted exemplarily in the lower trace.

The special focus is directed on the determination of the relative number of charges per molecule by measuring the amount of calcium adsorbed to lipid monolayers by monitoring low-energy β-radiation of ^45^Ca^2+^. The measurement of the adsorption of calcium to monolayers and also its displacement (Fig. 2) induced by the adsorption of various polycationic peptides and proteins is a technique which has been proven useful for studying the binding of pharmaceuticals to lipid monolayers [22-25].

In this paper, we used the adsorption of calcium to and displacement from lipid monolayers to characterize phospholipids and LPS monolayers and their interaction with a number of peptides/proteins. The results are compared with those obtained from calculations of the net negative charges on the basis of the chemical structure of the respective lipids, ζ-potential measurements and already published results obtained from innermembrane potential difference measurements using planar lipid bilayers [26]. The calculated number of charges per lipid molecule is linearly correlated with the adsorption of calcium.

## Results & discussion

### Determination of surface potentials of lipid aggregates

Charged residues in the headgroup of lipid molecules as well as attracted counter ions cause an electrical potential at the surface of the respective aggregates, the so-called Gouy-Chapman potential which decreases exponentially in the surrounding buffer (for review see [27]). Due to friction forces, aggregates moving in an externally applied electric field will loose weakly bound ions. The Gouy-Chapman potential at the interface between weakly and strongly bound ions, the plane of sheer, is called ζ-potential. From the velocity of the aggregates in an electric field, the electrophoretic mobility, the ζ-potential can be calculated via the Smoluchowski approximation [28].

**Figure 3:**
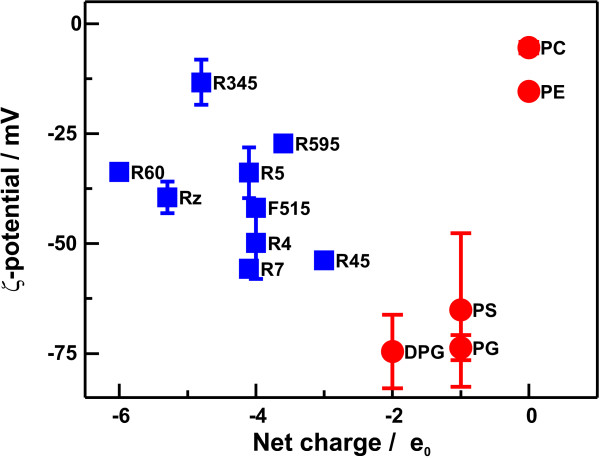
**Surface potential of lipid aggregates**. The ζ-potentials of aggregates made of LPS () or phospholipids () are plotted versus the net charge of the respective lipid molecules. ζ-potentials were measured in buffer containing 10 mM Tris, 2 mM CsCl_2 _and pH7. The lipid concentration was 0.01 mM. The net charges were calculated from the chemical structures determined by mass spectrometry.

To investigate the influence of lipid structures, in particular the presence of negatively charged groups, on the surface potential, we determined the electrophoretic mobility and calculated the respective ζ-potentials of various aggregates made from LPSs or phospholipids. The respective values are given in Tab. 1. In Fig. 3, the ζ-potential values for all lipids are plotted versus their net negative charges (calculated from the structure shown in Fig. 1, i.e., the amount of phosphate groups, Kdo's, 4-amino-4-deoxyarabinose (Ara4N), etc.) of the respective lipids.

**Table 1:** Properties of lipopolysaccharides and phospholipids used in this study. Given are the biochemical specifications of all lipids used in this study, the net charge calculated from mass spectrometric data, the results obtained from calcium adsorption experiments, given as the maximum β-intensity *I*_*max *_calculated according to Eq. 3, the electrophoretic mobilities μ, and the ζ-potential values. (n.d. – not determined, * – the values for phosphatidylcholine and phosphatidylglycerol could only be estimated)

**Lipopolysaccharid**						
**Species**	**Strain**	**LPS chemotype**	**calculated net charge/e_0_**	***I*_*max *_/cps**	**μ/10^-8 ^m^2 ^V^-1 ^s^-1^**	**ζ-potential/mV**

*S. minnesota*	R595	Re	- 3.6	265.8 ± 65.3	-2.13 ± 0.13	-27.2 ± 1.7
	R4	Rd_2_	- 4.0	345.3 ± 85.2	-3.90 ± 0,63	-49.9 ± 8.1
	R7	Rd_1_	- 4.1	307.9 ± 41.3	-4.35 ± 0.06	-55.7 ± 0.8
	Rz	Rd_1_	- 5.3	537.6 ± 69.2	-3.09 ± 0.28	-39.5 ± 3.6
	R5	Rc	- 4.1	307.9 ± 37.8	-2.65 ± 0.45	-33.9 ± 5.7
	R345	Rb_2_	- 4.8	545.9 ± 105.0	-1.04 ± 0.41	-13.3 ± 5.2
	R60	Ra	- 6.0	480.8 ± 91.6	-2.63 ± 0.03	-33.7 ± 0.4
*E. coli*	F515	Re	- 4.0	451.6 ± 119.7	-3.27 ± 0.12	-41.9 ± 1.5
*P. mirabilis*	R45	Re	- 3.0	265.8 ± 65.3	-4.21 ± 0.09	-53.9 ± 1.2

**Phospholipids**						

			**calculated net charge/e_0_**	***I*_*max*_/cps**	**μ/10^-8 ^m^2 ^V^-1 ^s^-1^**	**ζ-potential/mV**

phosphatidylglycerol			- 1.0	~60 ± 44.4*	-5.76 ± 0.23	-73.7 ± 2.9
phosphatidylserine			- 1.0	n.d.	-5.09 ± 1.37	-65.1 ± 17.5
diphosphatidylglycerol			- 2.0	182.4 ± 61.8	-5.83 ± 0.66	-74.6 ± 8.4
phosphatidylcholine			0	~0 ± 23.4*	-0.43 ± 0.10	-5.5 ± 1.3
phosphatidylethanolamine			0	n.d.	-1.20 ± 0.03	-15.4 ± 0.4

In the case of phospholipids, the absolute values of the ζ-potential increase linearly with increasing negative net surface charge as it could be proposed form the ζ-potential theory. The ζ-potential and the surface charge density of PG and DPG is almost the same, because the net charge as well as the surface area occupied by a DPG molecule is twice of that of a PG molecule. For the various LPSs used, we did not find any correlation between the calculated net charge and the ζ-potential. This might be explained by incorrect assumptions for the calculation of the ζ-potentials from the electrophoretic mobility. The determination of the ζ-potential is based on the Smoluchowski approximation [28] assuming a homogenously charged, smooth surface. In comparison to phospholipids, LPS molecules have relatively large headgroups with an oligosaccharide extending into the surrounding buffer. In addition, positively (Ara4N) and negatively charged groups (phosphate groups and Kdo's) are heterogeneously distributed in the lipid A part and the oligosaccharide.

Phospholipids are described to form multilamellar vesicles. Aggregates formed by LPS are also described to be multilamellar in the presence of di- or polyvalent ions at a temperature of 25°C [29,30]. It can be assumed that phospholipids and LPS vesicles are completely enclosed by bilayers under the conditions used in this study and, thus, the electrostatic properties of the outer leaflet of these vesicles are probably comparable with those of monolayers.

The surface potential profiles of LPS aggregates can be calculated according to the Gouy-Chapman theory [27] using the respective net charges (Tab. 1) and areas per LPS molecules which can be calculated from the amount of fatty acids (Tab. 2) [29]. The calculated values of the molecular areas have also been verified by determining the areas from pressure/area isotherms of monolayers. As shown by Pasquale et al [31], the planes of shear can be calculated from measured ζ-potentials and calculated surface potentials. The distances *d*_1 _between the aggregate surfaces and the planes of shear are in the range between 0.20 nm (LPS R7) and 0.42 nm (LPS R60) (Tab. 2). These calculated distances do not correlate with structural data, i.e. net charge and number of core sugars of the respective LPS, due to the fact that the model assumes smooth surfaces with homogeneous charge distributions.

**Table 2:** Calculated surface charge densities and planes of shear of the *S. Minnesota *LPS. The areas per molecule *A *were calculated according to Snyder et al [29], using the respective amounts of fatty acids determined from mass spectrometric data. The charge densities σ were calculated from *A *and the net negative charges given in Tab. 1. *d*_1 _and *d*_2 _represent the distances of the planes of shear from the aggregate surface taking into account 1 or 2 charged planes, respectively.

**Strain**	***A*/nm^2^**	**σ/e_0 _nm^-2^**	***d*_1_/nm**	***d*_2_/nm**
LPS R595	1.18	3.04	0.40	-
LPS R4	1.29	3.11	0.23	-
LPS R7	1.26	3.26	0.20	-
LPS Rz	1.05	5.04	0.36	1.26
LPS R5	1.26	3.26	0.35	-
LPS R345	1.19	4.02	0.24	1.04
LPS R60	1.04	5.75	0.42	1.38

Snyder et al. [29] concluded from electron density profiles of LPS R60 that its charges are located mainly in two distinct planes which are separated by a distance of 1.1 nm. The outer charged plane corresponds to the negatively charged phosphate groups linked to the heptoses in the core region of the LPS, the inner charged plane to the phosphate groups of the lipid A moiety. Using this model, we calculated the surface potential profiles of aggregates composed of those LPS having heptoses substituted with significant numbers of phosphate groups (LPS Rz, LPS R345, LPS R60) as superpositions of the Gouy-Chapman potential profiles Ψ_I _and Ψ_O _for the inner and the outer plane, respectively:

(Eq.1)Ψ_GC_(*x*) = Ψ_I _exp (- κ *x*) + Ψ_O _exp (- κ (*x *- 1.1 nm)) for *x *≥ 1.1 nm

where *x *is the distance from the inner charged plane and κ is the reciprocal Debye- length [27].

Ψ_GC _at the plane of shear corresponds to the ζ-potential. Using Eq. 1, the distance of the plane of shear *d*_2 _can be calculated according to:

(Eq. 2) *d*_2 _= - ln (ζ/[Ψ_I _+ Ψ_O _exp (κ 1.1 nm)])/κ.

The respective values for the *S. minnesota *LPS used in this study are given in Tab. 2. These results indicate that additional charges have a greater influence on the position of the plane of shear than an increased hydrodynamic drag resulting from additional saccharide groups. Studies on the influence of saccharides and charges of ganglioside G_M1 _[31,32] and PEG-PE [33] showed that the enhanced hydrodynamic drag of additional saccharides has a higher influence on the electrophoretic mobility of the respective particles than the amount and/or position of additional charges.

It should, however, be pointed out that our approach to describe the surface potential by the superposition of two distinct planes is only a simplified model. For example, the charges of the two phosphate groups of the lipid A and the two Kdo were assumed to be in one plane, however, the diglucosamine backbone of the lipid A moiety of the LPS is tilted with respect to the aggregate surface [34]. Thus, the charges are rather distributed in a volume than in a distinct plane.

Several advanced theories, taking a rough surface of the aggregate into account, have been published (for review see [35]). However, it should be mentioned that it is necessary to have detailed information on the 3D structures of the involved molecules. In case of a natural oligosaccharide, a defined 3D structure in solution has not been published so far, and because of a potential flexibility of oligosaccharides it is unlikely that structures can be determined which are precise enough for an accurate model to calculate surface potential profiles.

### Calcium adsorption to lipid monolayers

**Figure 4:**
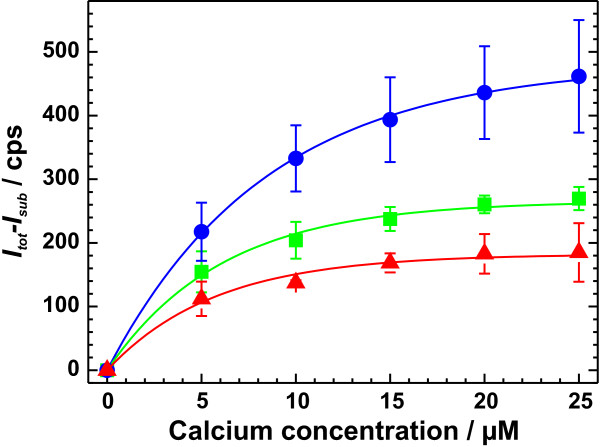
**Titration of calcium into the subphase underneath various monolayers**. Calcium was titrated into the subphase underneath various monolayers. The results are shown exemplarily for DPG (), LPS R45 (), and LPS R60 (). The data points represent the mean intensities determined from 3 independent experiments. The lines represent fits of the respective titration curves applying Eq. 3. Calcium adsorption was measured in a buffer solution containing 5 mM HEPES at pH7. The net charges were calculated from mass spectrometric data.

To investigate the adsorption of calcium to lipid monolayers made of various phospholipids and LPSs, calcium (doped with radioactive ^45^Ca^2+^) was titrated in amounts of up to 25 μM into the subphase underneath the monolayers composed of the identical number of the respective lipid molecules. The β-intensity was measured and plotted against the calcium concentration. To further analyze the data, the plots were fitted by the equation:

(Eq. 3) I(c)=Imax⁡(1−exp⁡(−cc0)),
 MathType@MTEF@5@5@+=feaafiart1ev1aaatCvAUfKttLearuWrP9MDH5MBPbIqV92AaeXatLxBI9gBaebbnrfifHhDYfgasaacH8akY=wiFfYdH8Gipec8Eeeu0xXdbba9frFj0=OqFfea0dXdd9vqai=hGuQ8kuc9pgc9s8qqaq=dirpe0xb9q8qiLsFr0=vr0=vr0dc8meaabaqaciaacaGaaeqabaqabeGadaaakeaacqWGjbqscqGGOaakcqWGJbWycqGGPaqkcqGH9aqpcqWGjbqsdaWgaaWcbaGagiyBa0MaeiyyaeMaeiiEaGhabeaakmaabmaabaGaeGymaeJaeyOeI0IagiyzauMaeiiEaGNaeiiCaa3aaeWaaeaacqGHsisldaWcaaqaaiabdogaJbqaaiabdogaJnaaBaaaleaacqaIWaamaeqaaaaaaOGaayjkaiaawMcaaaGaayjkaiaawMcaaiabcYcaSaaa@4607@

where *I(c) *is the β-intensity *I *as a function of the calcium concentration *c*, *I*_*max *_the maximum β-intensity at complete saturation of the lipid monolayer with calcium and *c*_0 _is the calcium concentration at which 1-1/e (~63%) of the monolayer is saturated with calcium. In Fig. 4, respective titration curves are shown exemplarily for DPG (red), LPS R45 (green), and LPS R60 (blue).

**Figure 5:**
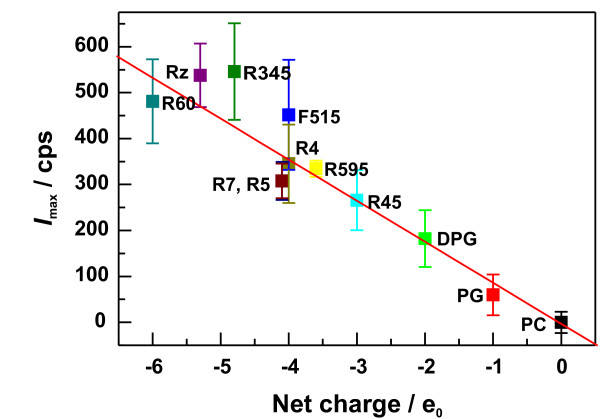
**Calcium adsorption to lipid monolayers**. The saturation values of calcium adsorption traces of lipid monolayers made of LPS or phospholipids are plotted versus the net charge of the respective lipid molecules. Calcium adsorption was measured in a buffer solution containing 5 mM HEPES at pH 7. The net charges were calculated from mass spectrometric data.

In Fig. 5, *I*_*max *_is plotted versus the net charge *q *of all lipids used for monolayer preparation. The respective values are given in Tab. 1. We found, that *I*_*max *_increases with increasing negative values of *q *and that *I*_*max *_and *q *are linear correlated with R = -0.98. The dependence of *I*_*max *_on *q *is given by *I*_*max *_= (-3.1 ± 21.0) cps + (-89.3 ± 6.5) cps/e_0 _*q*.

These results indicate that the amount of calcium adsorbed to a lipid monolayer depends solely on the number of negative charges and is not influenced by the 3D structure of the monolayer-forming lipid. Furthermore, it can be assumed from the linear correlation between *q *and *I*_*max *_that calcium ions intercalate into the oligosaccharide portion of the LPS, also binding directly to the charged groups of the lipid A part of the molecule. This is supported by calcium displacement experiments using fragments of rabbit and human cathelicidin peptide [36], in which some fragments were not able to displace 100% of the calcium adsorbed to the monolayer, indicating that some of the calcium ions are not accessible for the peptide.

### Influence of the lateral pressure on calcium adsorption

To check the influence of the lateral pressure on calcium adsorption, we used a Langmuir-Pockels film balance equipped with a movable barrier. LPS F515 monolayers were prepared and compressed to a final lateral pressure of 40 mN m^-1^, and the β-intensity was monitored.

**Figure 6:**
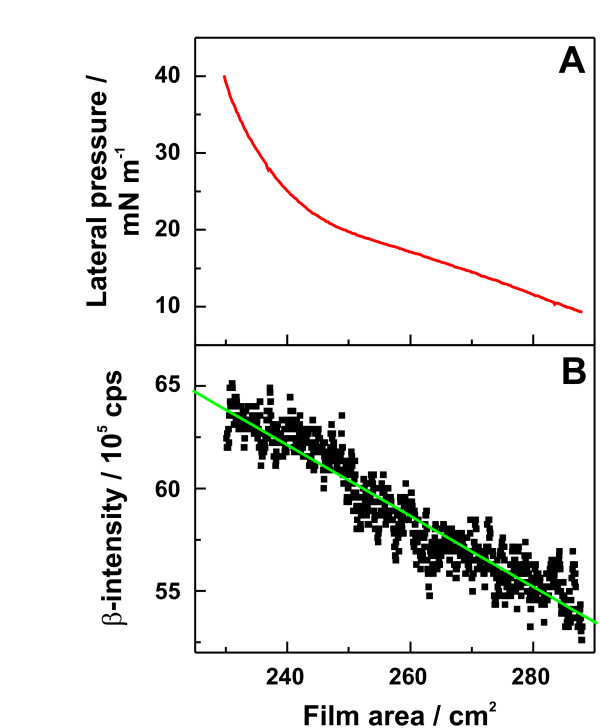
**Dependence of the β-intensity on area and lateral pressure of LPS F515 monolayers**. (A) Pressure/area isotherm of a LPS F515 monolayer. (B) Linear change of the β-intensity of ^45^Ca^2+ ^adsorbed on the monolayer determined under a constant area of the monolayer (green line: linear fit). Bathing solution: 12.5 μM calcium doped with radioactive ^45^Ca^2+ ^resulting in a relative β-activity of 250 Bq/ml, buffered with 5 mM HEPES, and adjusted to pH7.

In Fig. 6A, one representative pressure/area isotherm is shown. Up to ~20 mN m^-1^, the lateral pressure increases almost linearly with decreasing area, above 20 mN m^-1 ^the isotherm shows a typical non-linear dependence between area and lateral pressure. In contrast to the latter, the β-intensity increases linearly with decreasing film area (Fig. 6B). The increase of the β-intensity is due to the increased number of lipid molecules per unit of area and with that also below the detector. Thus, the amount of adsorbed calcium per molecule does not depend on the area per molecule or the lateral pressure in the monolayer, but solely on the number of molecular charge per unit of area.

### Calcium displacement by peptides/proteins

**Figure 7:**
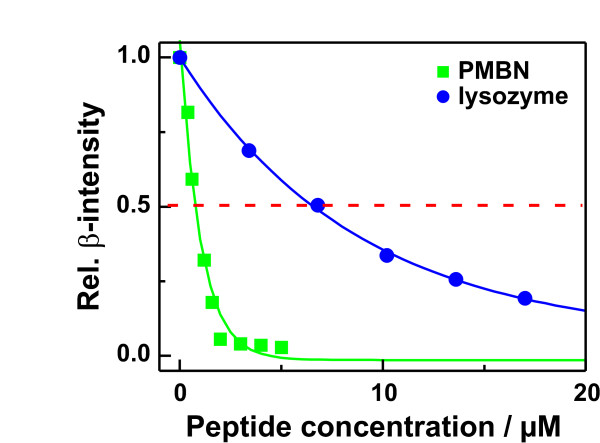
**Displacement of calcium from LPS F515 monolayers by polymyxin B nonapeptide and lysozyme**. Relative change of the β-intensity in dependence on peptide/protein concentration after addition of the nonapeptide of polymyxin B () or lysozyme () into the subphase underneath LPSF515 monolayers. Bathing solution: 12.5 μM Ca^2+ ^doped with radioactive ^45^Ca^2+ ^resulting in a relative β-activity of 250 Bq/ml, buffered with 5 mM HEPES, and adjusted to pH7 and the LPS F515 concentration was 9.6 nmol (160 nM).

To investigate the binding of various antimicrobial and LPS-neutralizing substances (Tab. 3) to lipid monolayers made from LPS, we performed calcium displacement experiments. Injections of increasing amounts of the peptides/proteins underneath the calcium saturated monolayers led to a decrease in the β-intensities due to a displacement of the calcium ions by the peptides/proteins. In Fig. 7, respective titration curves are given exemplarily for the nonapeptide of polymyxin B (PMBN) and lysozyme. The peptide PMBN led already at low micromolar concentrations to a strong decrease in β-intensity to almost the bulk level *I*_*sub*_, indicating a full displacement of calcium from the monolayer. In the case of lysozyme, the ability to displace calcium from the monolayer was significantly lower.

**Table 3:** IC_50 _calcium displacement values for various peptides/proteins. The molecular weights and the IC_50_-values (concentration of the peptides/proteins necessary to displace 50% of the calcium adsorbed to LPSF515 monolayers) are shown. Bathing solution: 12.5 μM calcium doped with radioactive ^45^Ca^2+ ^resulting in a relative β-activity of 250 Bq/ml, buffered with 5 mM HEPES, and adjusted to pH7; the LPSF515 concentration was 9.6 nmol (160 nM).

**Peptide/Protein**	**MW**	**IC_50_/μM**
rCAP18	3801	0.22
lactoferrin	78922	0.12
rBPI21	21063	0.16
PMB	1200	0.84
PMBN	963	0.76
lactalbumin	66000	1
lysozyme	14400	6.9
rHSA	66000	14
hemoglobin	64000	32

To compare the various peptides/proteins in their ability to displace calcium from an LPS monolayer, the IC_50 _values (the peptide concentration at which the β-intensity decreases to 50% of the saturation value) were calculated. The most potent substances were the proteins lactoferrin and the bactericidal/permeability-increasing protein (rBPI21) and a fragment of the 18 kDa rabbit cationic antimicrobial protein (rCAP18_106–137_) which were active already at submicromolar concentrations. The IC_50 _values of the recombinant human serum albumin (rHSA) and hemoglobin were by two orders of magnitude higher. Interestingly, rCAP18, PMB, BPI, and lactoferrin are well-known LPS-neutralizing peptides [37]. In contrast to this, rHSA and hemoglobin do not inhibit the LPS-induced activation of human mononuclear cells [38], in case of hemoglobin the TNFα production is even increased [39]. Thus, the ability of the peptides/proteins to displace calcium from LPS monolayers seems to be correlated with their ability to neutralize LPS.

Divalent cations lead to a cross linking between the LPS individual molecules [40] and lead, therefore, to an increased binding energy within the aggregate surface. Probably, the polyvalent peptides/proteins lead to a binding energy, which is higher than that provoked by divalent cations such as Mg^2+ ^or Ca^2+^. Thus, the binding of LPS to proteins being involved in the LPS-induced activation of mononuclear cells is inhibited.

In many cases, the intercalation of peptides/proteins decreases with increasing lateral pressure. Therefore, to guarantee a maximal interaction between the monolayers and the peptides/proteins, experiments were done at a relatively low lateral pressure as compared to that in a natural cell membrane which is discussed to be in the range from 20 to 30 mN m^-1 ^[41,42].

**Figure 8:**
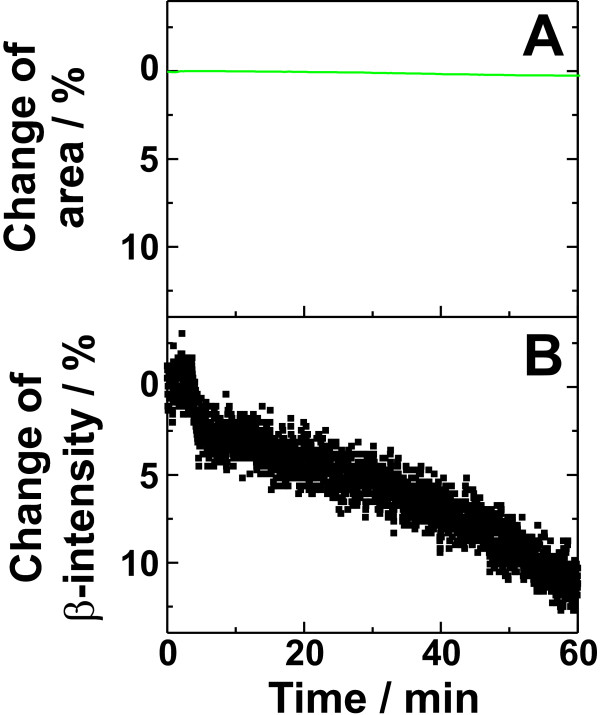
**Influence of polymyxin B on the area of a monolayer and the adsorbed calcium**. Injection of polymyxin B (350 μM) underneath a LPS F515 monolayer at a constant lateral pressure of 27 mN m^-1 ^led to (A) no change of the area of the monolayer (B) a decrease of the calcium adsorbed on the monolayer. Bathing solution: 12.5 μM Ca^2+ ^doped with radioactive ^45^Ca^2+ ^resulting in a relative β-activity of 250 Bq/ml, buffered with 5 mM HEPES, and adjusted to pH7.

In the next step of our experiments we investigated the ability of PMB to bind to LPS monolayers without intercalating into it at high lateral pressures. When 500 μl of a 350 μM PMB solution were injected into the subphase (250 ml) underneath an LPSF515 monolayer at a constant lateral pressure of 27 mN m^-1^, no significant change of the film area was observed (Fig. 8A), indicating that PMB does not intercalate into the lipid matrix at this lateral pressure. However, it led to a decrease in β-intensity of about 10% within 60 min after peptide addition (Fig. 8B), demonstrating that PMB, even if it is not able to intercalate into the monolayer and increase its surface area, it is able to bind to the monolayer surface and to displace calcium. Thus, the calcium displacement method is a helpful complementation to characterize the interaction between lipid monolayers and peptides/proteins, because it is now possible to differentiate between intercalation and accumulation of peptides.

## Conclusion

The characterization of the electrical potential of lipid membranes and of electrostatically driven interactions between lipids and peptides/proteins are important aims for an understanding of the molecular interaction mechanisms. The adsorption of calcium ions to lipid monolayers can be determined by measuring the β-intensity of ^45^Ca^2+ ^which is linearly proportional to the calculated net negative charge of the respective phospholipids or LPSs. Thus, calcium ions seem to bind to all negatively charged groups of LPS, even to those which are located close to the LPS backbone. Larger probe molecules might not be able to reach these groups. The peptide/protein-induced displacement of calcium ions is a usable parameter for the determination of the adsorption of molecules to lipid monolayers. The film balance experiments provide information on i) the adsorption of peptides at the monolayer surface caused by electrostatic interactions determined by calcium displacement and ii) the intercalation into the monolayer caused by electrostatic and hydrophobic interactions. In contrast to this, in calorimetric and surface plasmon resonance experiments it is difficult or in many cases not possible to distinguish between binding and intercalation.

The determination of the ζ-potential provides valuable information for the characterization of the electrostatic interaction between peptides/proteins and phospholipid or LPS aggregates. However, due to the complexity of the LPS molecules, the theories, which are based on the assumption of flat surfaces, fail to describe the potential of LPS aggregates. The calculation of the respective planes of shear demonstrates that the ζ-potentials depend on the number of saccharides and charged groups. As we showed earlier [43], the innermembrane potential difference, which can be determined from experiments using the inner-field compensation method [20] or the carrier transport by nonactin, also strongly depends on the thickness of the phospholipid or LPS headgroup regions.

It has been shown in many publications that the intercalation of peptides/proteins into lipid monolayers and bilayers can be different. However, according to Schoch et al. [21,44] the electrostatic potential of the leaflet of the bilayer presented to the buffer is the same as that of the respective monolayer. Therefore, the peptide/protein-induced displacement of calcium ions from various monolayers and reduction of the ζ-potential of aggregates are both based on the same electrostatic properties of the lipid matrices.

In summary, measurements of calcium adsorption and displacement provide additional useful information for the characterization of the electrostatic interaction between negatively charged monolayers and peptides/proteins.

## Methods

### Lipids and peptides/proteins

Rough mutant LPS from *Salmonella enterica *serovar Minnesota (*S. minnesota*) strains R595 (LPS R595), R4 (LPS R4), R7 (LPS R7), Rz (LPS Rz), R5 (LPS R5), R345 (LPS R345), and R60 (LPS R60) as well as deep rough mutant LPS from *Escherichia coli *strain F515 (LPS F515) and *Proteus mirabilis *strain R45 (LPS R45) were used [45-50]. LPS was extracted by the phenol/chloroform/petroleum ether method [51], purified, lyophylized, and transformed into the triethylamine salt form. The chemical structures are given in Fig. 1. The amounts of nonstoichiometric substitutions by fatty acids (data not shown), Ara4N, additional phosphates, and phosphoethanolamine were analyzed by mass spectrometry. In LPS R595, the Ara4N linked to the 4'-phosphate was present at a level of 40%. In LPS R45, approximately 50% of the 4'-phosphate of lipid A and 50% of the first Kdo were substituted with Ara4N. By taking into account the amounts of negatively charged phosphate groups and Kdo's and the positively charged Ara4Ns the net charges of the LPSs as summarized in Tab. 1, were calculated.

Phosphatidylcholine (PC) from egg yolk lecithin, phosphatidylglycerol (PG) from egg yolk lecithin (sodium salt), and synthetic diphosphatidylglycerol (DPG) were purchased from Avanti Polar Lipids (Alabaster, AL, USA) and used without further purification.

Polymyxin B (PMB) and its nonapeptide (PMBN), lactoferrin from human milk, lactalbumin from bovine milk, lysozyme from chicken egg white, and human hemoglobin were purchased from Sigma Aldrich (Deisenhofen, Germany), and recombinant human serum albumine (rHSA) from Welfide Corporation (Osaka, Japan). Recombinant BPI_1–193 _(rBPI21) was a kind gift of XOMA (Berkeley, CA, USA). The rabbit CAP18_106–137 _was a kind gift provided by J.W. Larrick (Palo Alto Institute of Molecular Medicine, Mountain View, CA, USA).

### Determination of the surface potential

Fixed charges within the headgroups of lipid molecules cause an electric potential at the lipid bilayer surface with respect to the surrounding bathing solution, the surface potential [27,52]. The ζ-potential is related to the surface potential of the lipid aggregates, and can be calculated from the electrophoretic mobility of the aggregates according to the Smoluchowski approximation [53]:

(Eq. 4) ζ = μ η/(ε_r _ε_0_)

where ζ is the ζ-Potential, μ the electrophoretic mobility, η the viscosity (0.89·10^-3 ^kg m^-1^s^-1^), ε_0 _the dielectric constant of vacuum (8.854·10^-12 ^A^2^s^4^kg^-1^m^-3^) and ε_r _the dielectric permittivity of water (78.54).

To study the surface potential of phospholipid and LPS aggregates, we determined their ζ-potentials. The measurements were performed on a ZetaSizer4 (Malvern Instruments GmbH, Herrsching, Germany) at 25°C and with a driving electric field of 19.2 V cm^-1^.

Aggregates were prepared as 1 mM aqueous dispersions of lipid in buffer (10 mM Tris, 2 mM CsCl_2_, pH7). Briefly, the lipid dispersions were sonicated for 20 min at 60°C, cooled down to 4°C for 30 min and temperature-cycled twice between 60°C and 4°C (30 min each). The dispersions were equilibrated overnight at 4°C. Prior to ζ-potential measurements, lipid dispersions were diluted to a final concentration of 0.01 mM. Presented values (Fig. 3) are mean values with standard derivations resulting from 3 to 5 independent experiments.

### Preparation of lipid monolayers used in film balance experiments

In general, two types of Langmuir-Pockels film balance experiments were utilized: (i) at a constant and (ii) at a variable film area. In both types of experiments the phospholipids were dissolved in chloroform and the LPSs in a 10:1 (v:v) mixture of chloroform and methanol at a concentration of 1 mM. All lipids were deposited in the given amounts on the subphase and the solvents were allowed to evaporate for 5 min.

### Determination of calcium adsorption to and displacement from lipid monolayers

Calcium adsorption to lipid monolayers prepared from phospholipids or LPSs was determined by depositing 10 nmol of the respective lipid on 60 ml of an aqueous subphase buffered with 5 mM HEPES and adjusted to pH7. After equilibration of the monolayer, 5 aliquots of 200 μl of a 1.5 mM calcium solution adjusted to a relative β-activity of 24 kBq/ml with radioactive ^45^Ca^2+ ^(Amersham Buchler, Braunschweig, Germany) were added to the subphase, resulting in a final calcium concentration of 25 μM. Calcium ions bind to the negatively charged headgroups of the lipids, and the low-energy β-radiation originating from the bound ^45^Ca^2+ ^is not absorbed by the hydration shell anymore and, therefore, an increase in β-intensity was observed using a β-counter (gas ionization detector LB124, Berthold, Wildbad, Germany) (Fig 2, step 1 & 2). Thus, this method allows the determination of the relative amount of calcium bound to the monolayer [24]. To keep the number of lipid molecules and, therefore, the potential number of binding sites for calcium underneath the detection area of the β-counter constant, the experiments were performed in an acrylic glass trough with a total constant film area of 112 cm^2^.

The β-intensity *I*_*mono *_originating from ^45^Ca^2+ ^bound to the lipid monolayer at a given Ca^2+ ^concentration was calculated according to the equation:

(Eq. 5) *I*_*mono *_= *I*_*tot *_- *I*_*sub*_,

where *I*_*tot *_is the β-intensity originating from the monolayer and the subphase and *I*_*sub *_is the β-intensity of the pure subphase. These and the following experiments were performed at a subphase temperature of 20°C instead of 37°C to avoid condensation at the β-counter.

To investigate the capacity of various peptides/proteins to displace divalent Ca^2+ ^ions from LPS F515 monolayers, a subphase containing 12.5 μM Ca^2+ ^doped with radioactive ^45^Ca^2+ ^(resulting in a relative β-activity of 250 Bq/ml), buffered with 5 mM HEPES at pH7 was used. Also in the displacement experiments, the number of LPS molecules was kept constant. The agents were added to the subphase at a constant total film area and relatively low lateral pressure of the monolayer (~10 mN m^-1^). This way, the final pressure after saturation of the intercalation of agents was still in the range of lateral pressures in biological membranes [41,42]. The peptides/proteins were added to the subphase in different concentrations, and the equilibrium β-counting rates were recorded (Fig. 2, step 3). The relative *I*_*rel*_(*c*) in dependence on the peptide/protein concentration *c *was calculated from

(Eq. 6) 

where *I*_*tot*_(*c*) is the absolute β-intensity, *I*_*sub *_the β-intensity originating from the pure subphase, and *I*_*mono *_the β-intensity originating from the monolayer. From the displacement curves the concentrations at which 50% of the calcium were displaced (IC_50 _value) by the peptides/proteins were determined and summarized in Tab. 1.

All presented values are mean values with standard derivations resulting from 3 to 4 independent experiments.

### Influence of the lateral pressure on the adsorption of calcium to LPS monolayers

A Langmuir-Pockels film balance equipped with a Wilhelmy system (Munitech, München, Germany) was used to determine the lipid pressure/area isotherms of LPS F515 monolayers. The lateral pressure, area, and the β-intensity were determined. For the experiments, 36 μl of the LPS F515 were spread on the buffer surfaces of 290 cm^2^. The solvent was allowed to evaporate at zero pressure for 5 min. Then the monolayers were isothermally compressed at a speed of 1.5 mm^2 ^s^-1 ^to a lateral pressure of 40 mN m^-1^.

## Abbreviations

AMP – antimicrobial peptides

Ara4N – 4-amino-4-deoxyarabinose

BPI – bactericidal/permeability increasing protein

DPG – diphosphatidylglycerol

ENP – endotoxin-neutralizing protein

Kdo – 3-deoxy-alpha-D-manno-oct-2-ulosonic acid

LPS – lipopolysaccharide

LTA – lipoteichoic acid

OM – outer membrane

PC – phosphatidylcholine

PE – phosphatidylethanolamine

PEG – poly(ethylene glycol)

PG – phosphatidylglycerol

PMB – polymyxin B

PMBN – nonapeptide of PMB

PS – phosphatidylserine

rBPI21 – recombinant 21 kDa fragment of BPI

rCAP18 – 18 kDa rabbit cationic antimicrobial protein

rHSA – recombinant human serum albumin

## Authors' contributions

SOH designed and carried out the adsorption binding assay and drafted the manuscript. MUH carried out the ζ-potential experiments and helped to draft the manuscript. AW participated in the film balance experiments. US participated in the design of the study and helped to draft the manuscript. TG participated in the design and coordination of the study, performed some of the monolayer experiments and helped to draft the manuscript. All authors read and approved the final manuscript.
